# Identifying ureteral stent encrustation using machine learning based on CT radiomics features: a bicentric study

**DOI:** 10.3389/fmed.2023.1202486

**Published:** 2023-08-02

**Authors:** Junliang Qiu, Minbo Yan, Haojie Wang, Zicheng Liu, Guojie Wang, Xianbo Wu, Qindong Gao, Hongji Hu, Junyong Chen, Yingbo Dai

**Affiliations:** ^1^Department of Urology, Fifth Affiliated Hospital of Sun Yat-sen University, Zhuhai, Guangdong, China; ^2^Department of Radiology, Fifth Affiliated Hospital of Sun Yat-sen University, Zhuhai, Guangdong, China; ^3^Department of Urology, Zhuhai People’s Hospital (Zhuhai Hospital Affiliated with Jinan University), Zhuhai, Guangdong, China

**Keywords:** ureteral stent encrustation, radiomics, machine learning, artificial intelligence, nomograms

## Abstract

**Obstructive:**

To develop and validate radiomics and machine learning models for identifying encrusted stents and compare their recognition performance with multiple metrics.

**Methods:**

A total of 354 patients with ureteral stent placement were enrolled from two medical institutions and divided into the training cohort (*n* = 189), internal validation cohort (*n* = 81) and external validation cohort (*n* = 84). Based on features selected by Wilcoxon test, Spearman Correlation Analysis and least absolute shrinkage and selection operator (LASSO) regression algorithm, six machine learning models based on radiomics features were established with six classifiers (LR, DT, SVM, RF, XGBoost, KNN). After comparison with those models, the most robust model was selected. Considering its feature importance as radscore, the combined model and a nomogram were constructed by incorporating indwelling time. Accuracy, sensitivity, specificity, area under the curve (AUC), decision curve analysis (DCA) and calibration curve were used to evaluate the recognition performance of models.

**Results:**

1,409 radiomics features were extracted from 641 volumes of interest (VOIs) and 20 significant radiomics features were selected. Considering the superior performance (AUC 0.810, 95%CI, 0.722–0.888) in the external validation cohort, feature importance of XGBoost was used as a radscore, constructing a combined model and a nomogram with indwelling time. The accuracy, sensitivity, specificity and AUC of the combined model were 98, 100, 97.3% and 0.999 for the training cohort, 83.3, 80, 84.5% and 0.867 for the internal cohort and 78.2, 76.3, 78.8% and 0.820 for the external cohort, respectively. DCA indicates the favorable clinical utility of models.

**Conclusion:**

Machine learning model based on radiomics features enables to identify ureteral stent encrustation with high accuracy.

## Introduction

A ureteral stent is placed commonly in most cases of lithotripsy and sometimes other urological operations. It plays a crucial role in supporting the ureter and assisting the outflow of urine from the kidney to the bladder (1). Now, ureteral stent placement is considered a standard and irreplaceable urological method. Even so, some potential side effects of placement of ureteral stent can not be ignored, such as discomfort, hematuria, malposition, urinary tract infection, encrustation, and so on (2).

Encrustation is associated chiefly with indwelling time. El-Faqih et al. (3) found that in patients with ureteral stent placement, the risk of encrustation is 9.2, 47.5 and 76.3% for 6 weeks, 6 to12 weeks and longer than 12 weeks before removal, respectively. Some side effects could happen to patients with ureteral stent encrustation, such as obstruction, infection, pain, lower urinary tract symptoms (LUTS), and especially removal failure.

Changes in physical properties of stents are the pivotal reason of removal failure. Encrustation prevention strategies such as urine pH modulation, novel materials and stent coatings, say, hydrophilic biodegradable stent and Silicone hydrophilic coating, made little difference in medical practice (4–9). Once stents get encrusted, they become calcified and brittle, losing their tensile strength (1, 2). Blind removal of large-volume encrustation stents raises the risk of fracturing the stent and avulsing the ureter (2, 10). These issues impair the quality of treatment and impose an additional financial burden on patients. Multiple extra procedures are needed to deal with stent encrustation, giving rise to up to 16% of lawsuits in the endourology field (11). However, it is still challenging to identify encrustation in a non-invasive way.

Severe encrustation can be recognized on CT or standard KUB x-ray before removal, but inconspicuous encrustation is usually missed in clinical practice (12). Though Acosta-Miranda and Arenas proposed several score systems to evaluate the severity of encrustation and predict the consequence of encrusted ureteral stent taking out, all of these depend on the premise that encrustation was confirmed. The emergence of radiomics provides a new direction for identifying ureteral stent encrustation.

Radiomics is a powerful method that assists physicians in making the most accurate diagnosis and predicting the prognosis and outcomes by further mining and analyzing massive data (13–15). The procedures refer to the high-throughput extraction of a mass of data from images (CT, MR, PET-CT, etc.) to achieve volumes of interest (VOIs) segmentation, feature extraction and model establishment (16). Regarding urolithiasis, radiomics wonderfully differentiated kidney stones from phleboliths, recognizing infection stones, calcium oxalate monohydrate stones, etc. (17–20). Nevertheless, it has yet to be put into identifying ureteral stent encrustation.

Therefore, in this study, we tend to establish a model with high accuracy in identifying ureteral stent encrustation using machine learning based on CT radiomics features. Early identification and prevention of low or median degrees of encrustation is one of the most effective measures to treat the complications of encrustation. The ultimate combined model could facilitate clinical decision-making and benefit patients.

## Methods

### Patients population

The retrospective study was approved by the Ethics Review Committee of the Fifth Affiliated Hospital of Sun Yat-sen University, and the requirement for informed consent was waived concurrently. A total of 354 patients underwent endoscopic lithotripsy and placement of ureteral stent from the Fifth Affiliated Hospital of Sun Yat-sen University and Zhuhai People’s Hospital (Zhuhai Hospital Affiliated with Jinan University) were enrolled with the following criteria. Among them, 270 patients treated in the Fifth Affiliated Hospital of Sun Yat-sen University (SYSU5H) from June 2016 to January 2023 were allocated randomly to the training cohort (*n* = 189) and internal validation cohort (*n* = 81) in a ratio of 7:3 and 84 patients treated in Zhuhai People’s Hospital (ZPH) between June 2017 and January 2023 were assigned to the external validation cohort. The inclusion criteria: (1) patients underwent endoscopic lithotripsy and placement of ureteral stent, (2) ureteral stent was removed successfully, and encrustation around stent is visible to the naked eye, (3) patients had undergone preoperative non-contrast CT scan before ureteral stent removal. The exclusion criteria: (1) placement of a ureteral stent for other reasons except for endoscopic lithotripsy, (2) non-contrast CT images with severe damage or noticeable artifact, (3) the location of ureteral stent encrustation is hard to distinguish; (4) severe displacement of the ureteral stent or malformation of the urinary system.

Baseline data, like sex, age, surgery and indwelling time, were collected from the medical record. The severity of encrustation was evaluated by the forgotten encrusted calcified score (FECal), the kidney ureter bladder score (KUB), and the visual grading for ureteral encrusted stent classification (V-GUES) (21, 22). Each removal encrusted stent was collected into a sample database. 101 (37.4%) and 32 (38.1%) patients have encrusted stents in SYSU5H and ZPH, respectively. Details of patients are shown in Table 1.

**Table 1:** Baseline characteristics of the patients.

Characteristic	SYSU5H		*p*	ZPH		*p*
	Non-encrustation	Encrustation		Non-encrustation	Encrustation	
Sex (%)						
Male	100 (59.2)	62 (61.4)	0.719	36 (69.2)	15 (46.9)	0.042
Female	69 (40.8)	39 (38.6)		16 (30.8)	17 (53.1)	
Age, yr	51	52	0.464	45	58	0.008
(median [IQR]) encrustation location(%)	[40.00, 60.00]	[44.00, 63.00]		[39.00, 54.00]	[42.00, 67.25]	
B	0 (0.0)	24 (23.8)		0 (0.0)	11 (34.4)	
K	0 (0.0)	4 (4.0)		0 (0.0)	0 (0.0)	
KB	0 (0.0)	7 (6.9)		0 (0.0)	0 (0.0)	
KUB	0 (0.0)	1 (1.0)		0 (0.0)	1 (3.1)	
U	0 (0.0)	60 (59.4)		0 (0.0)	18 (56.2)	
UB	0 (0.0)	5 (4.9)		0 (0.0)	2 (6.3)	
KUB score		2.65 ± 0.99			2.25 ± 1.41	
FECal grade(%)						
Grade 1	0 (0.0)	68 (67.3)		0 (0.0)	28 (87.5)	
Grade 2	0 (0.0)	21 (20.7)		0 (0.0)	1 (3.1)	
Grade 3	0 (0.0)	4 (4.0)		0 (0.0)	2 (6.3)	
Grade 4	0 (0.0)	7 (7.0)		0 (0.0)	0 (0.0)	
Grade 5	0 (0.0)	1 (1.0)		0 (0.0)	1 (3.1)	
V-GUES classification(%)						
Class A	0 (0.0)	21 (20.8)		0 (0.0)	29 (90.6)	
Class B	0 (0.0)	65 (64.3)		0 (0.0)	2 (6.3)	
Class C	0 (0.0)	14 (13.9)		0 (0.0)	0 (0.0)	
Class D	0 (0.0)	1 (1.0)		0 (0.0)	1 (3.1)	
Surgery (%)						
PCNL	36 (21.3)	20 (19.8)	0.446	17 (32.7)	3 (9.4)	0.044
RIRS	59 (34.9)	29 (28.7)		17 (32.7)	16 (50.0)	
URL	74 (43.8)	52 (51.5)		18 (34.6)	13 (40.6)	
Indwelling time, *d*	28	35	<0.001	28	28	0.079
(median [IQR])	[27.00, 32.00]	[28.00, 55.00]		[27.75, 31.00]	[26.00, 29.00]	

SYSU5H, the Fifth Affiliated Hospital of Sun Yat-sen University; ZPH, Zhuhai People’s Hospital (Zhuhai Hospital Affiliated with Jinan University), IQR, interquartile range.

### CT data acquisition and standardization

All patients underwent preoperative non-contrast CT scans ranging at least from the upper edge of the kidney to the lower edge of the sciatic tuberosity before removal. Patients enrolled from SYSU5H performed on one of the following two scanners: SOMATOM DEFINITION FLASH, UCT 640. Acquisition parameters for all SYSU5H images were as follows: real-time exposure dose auto-adjustment, reference tube voltage, 100 kV; 0.9 beam pitch; automated varied milliampere-second settings; collimation width, 5 mm, layer thickness 2 mm. Patients enrolled from ZPH performed on one of the following five scanners: GE Revolution Maxima, SOMATOM DEFINITION FLASH, UCT 780, SOMATOM PERSPECTIVE, and GE Revolution. Acquisition parameters for all ZPH images were as follows: real-time exposure dose auto-adjustment, reference tube voltage, 120–140 kV; 1.0 beam pitch; automated varied milliampere-second settings; collimation width, 5 mm, layer thickness 1 mm/1.25 mm. All images were collected in DICOM format from the picture archiving and communication system (PACS).

The information on CT instruments and parameters varied among different hospitals, which exists potential batch differences. Principal component analysis (PCA) was used to visualize the batch effect of radiomics features, and the ComBat algorithm was used for batch correction. Considering the imbalance of the data set and the difference in CT images, upsampling analysis was applied to improve the data quality. Details of standardization are shown in Supplementary material S1.

### Feature extraction and selection

Two urologists with >10 years of diagnostic experience were involved in delineating the volume of interest (VOI) manually layer by layer. Manual segmentation and relative radiomics feature extraction were both achieved in the Radcloud platform.^1^ Any discrepancy was addressed by discussion. Then all contours were reviewed by the senior radiologists and urologists. Finally, 641 VOIs were segmented from 354 patients’ images which were applied to the subject analysis.

**Figure 1:**
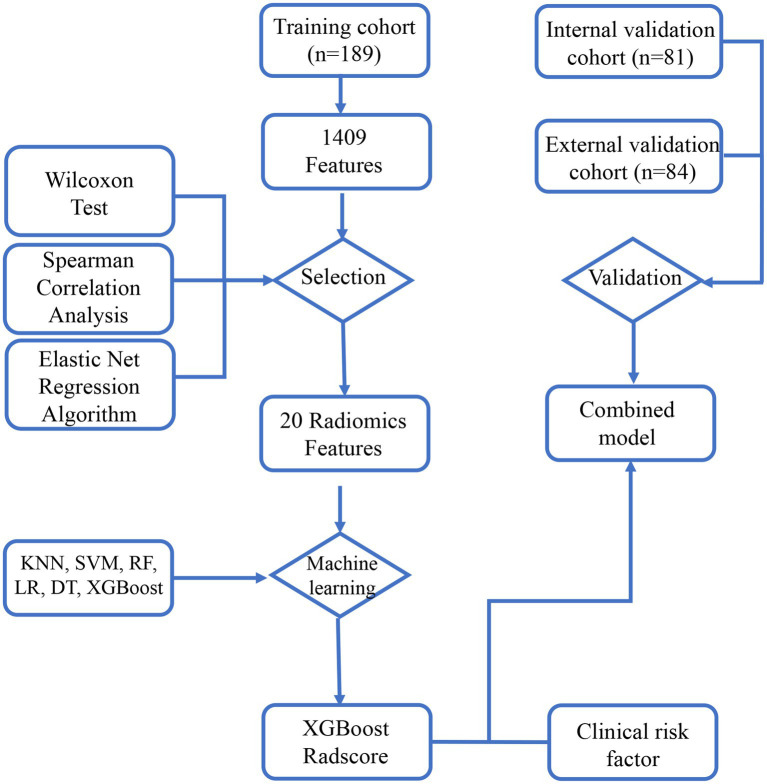
The study flowchart. The flowchart describes the successive study steps. LASSO, least absolute shrinkage and selection operator; KNN, k-NearestNeighbor; SVM, Support Vector Machine; XGBoost, Xtreme Gradient Boosting; RF, Random Forest; LR, Logistic Regression; DT, Decision tree.

**Figure 2:**
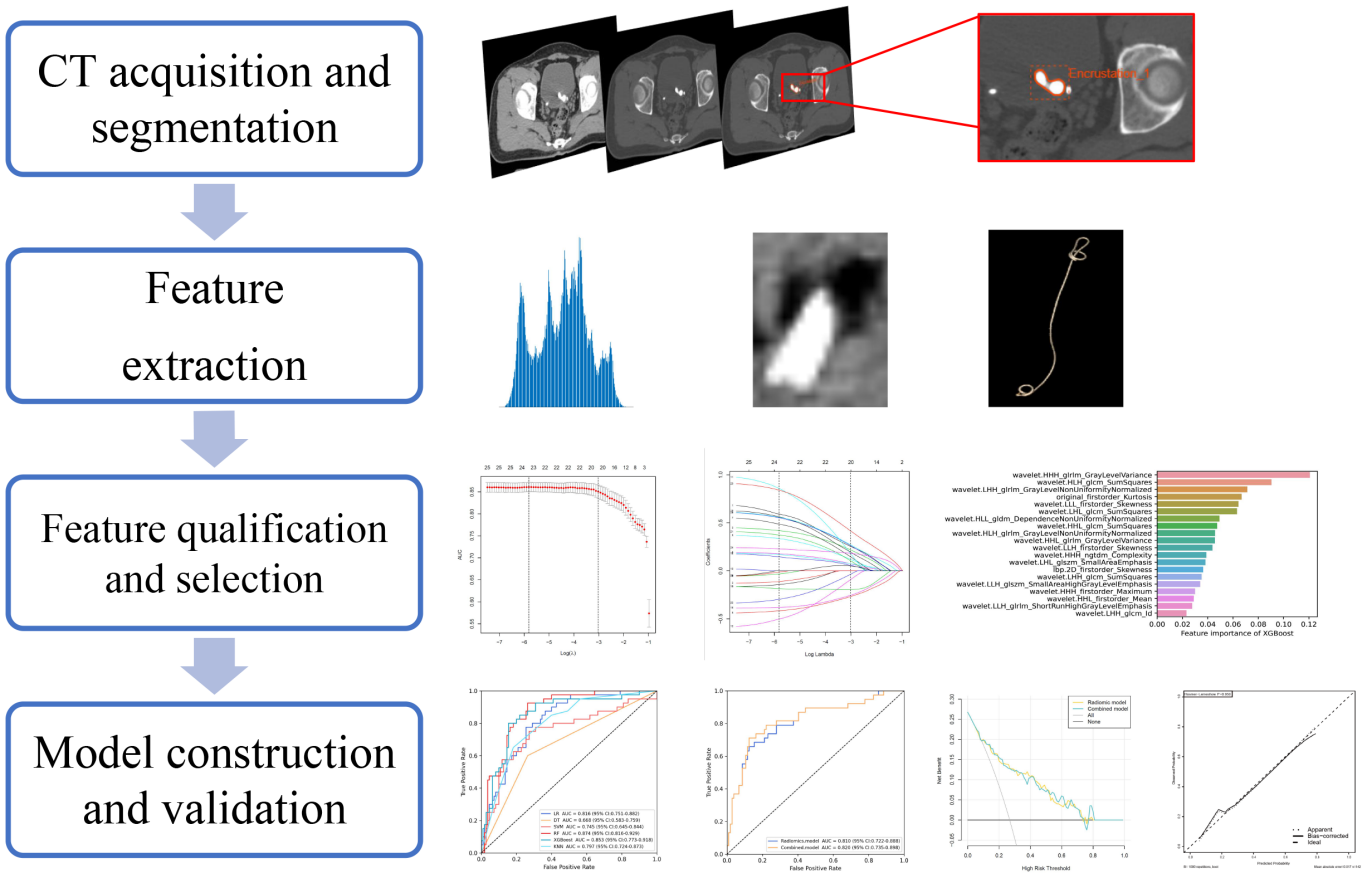
The radiomics workflow. The radiomics workflow illustrates the radiomics procedure.

**Figure 3:**
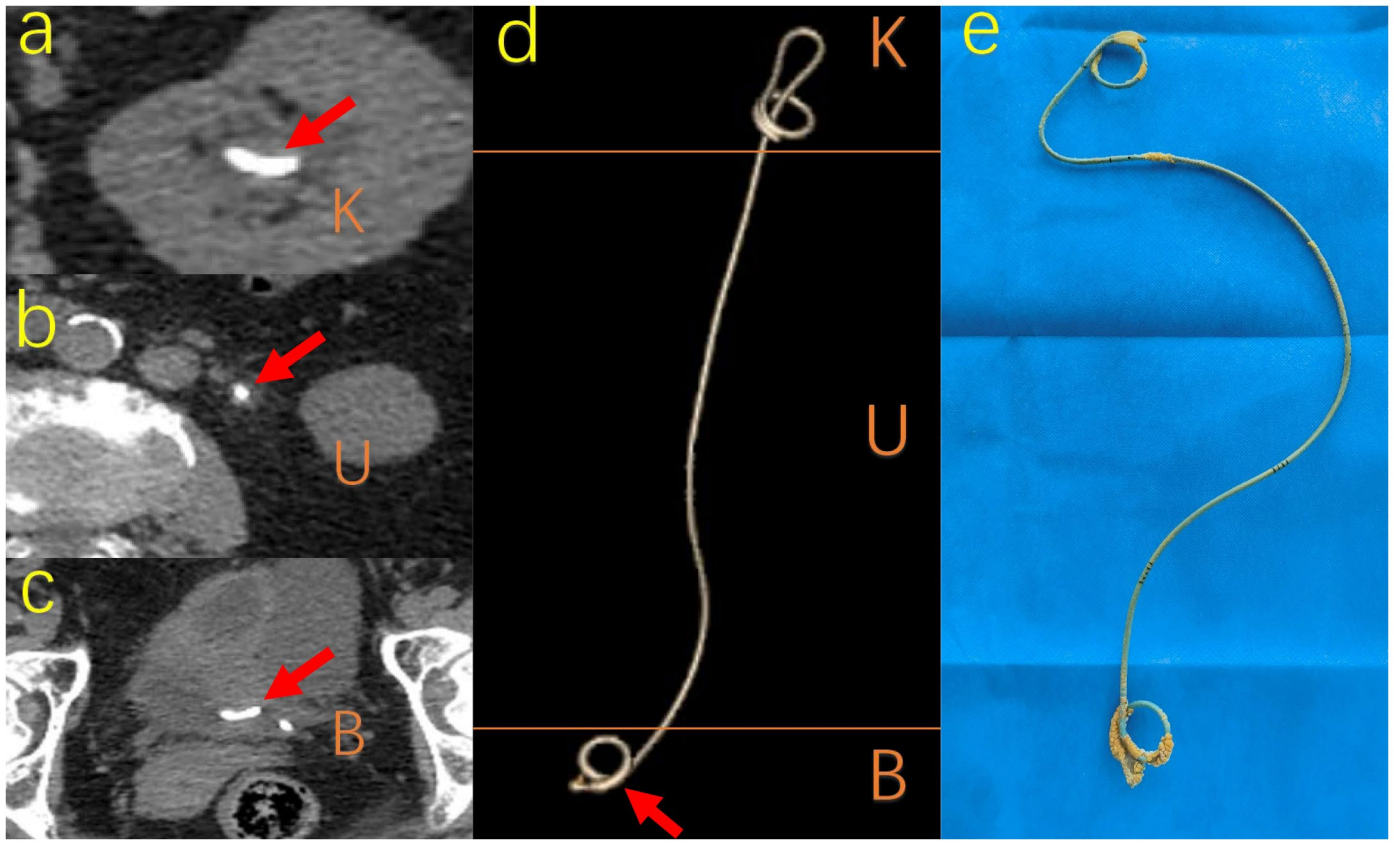
Different visual manifestations of encrustation. A 69-year-old woman with ureteral stent placement for 322 days forgot to remove it so that typical encrustations surrounded on K, U, B segments, respectively. **(A)** **(B)** **(C)** shows CT image pattern of encrustation on different segments respectively and **(D)** shows the CT image 3D reconstruction of the whole ureteral stent. **(E)** The actual sample removed from the patient shows the actual shape and location of encrustations. K, kidney; U, ureter; B, bladder; CT, computed tomography.

**Figure 4:**
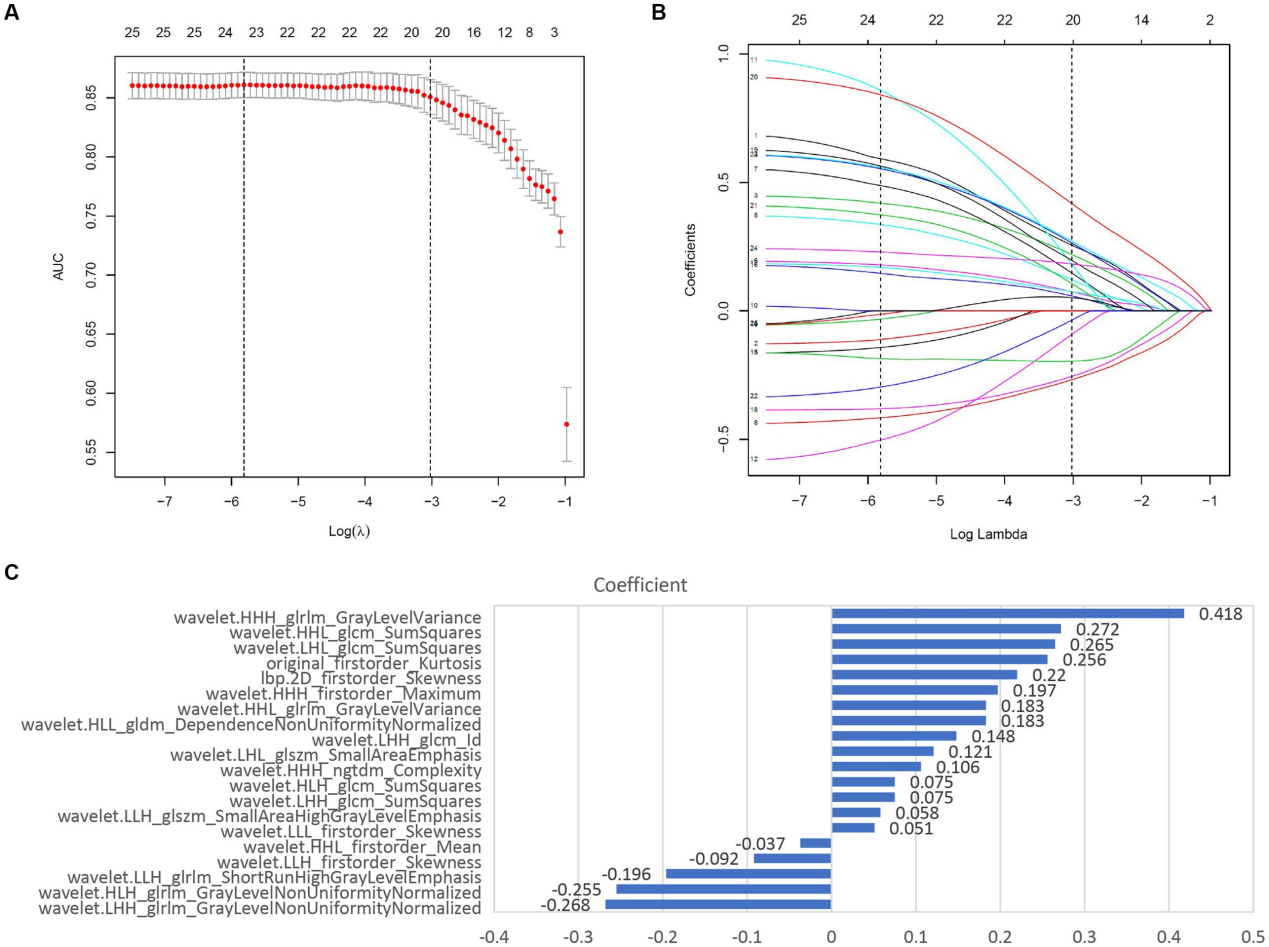
Screening and performance of radiomics features using LASSO. **(A)** Selection of the tuning parameter (λ). The LASSO logistic regression model was used with penalty parameter tuning conducted by 10-fold cross-validation. The AUC was plotted versus log(λ). The vertical dotted line is plotted at the optimal λ value. The optimal AUC with log(λ) value of -3.022 was selected. **(B)** LASSO coefficient profiles of the 25 radiomics features selected by Wilcoxon test and Spearman Correlation Analysis. The vertical dotted line was plotted at the log(λ) value of -3.022, resulting in 20 nonzero coefficients. **(C)** Histogram shows the coefficients of the selected features in the radiomics signature. GLCM, gray level co-occurence matrix; GLDM, gray level dependence matrix; GLSZM, gray level size zone matrix; NGTDM, neighboring gray tone difference matrix; GLRLM, Gray-Level Run-Length Matrix.

The study flowchart and radiomics workflow are shown in Figures 1 and 2. A total of 1,409 quantitative imaging features were extracted. First, Wilcoxon test analyzed the significance of the radiomics features, and 590 correlation features were obtained (*p* < 0.05 was considered significant; Figure 3). Then, Spearman Correlation Analysis was performed on the above radiomics features. These features whose threshold exceeds 0.6 were defined as redundancy and deleted, and 25 features remained. Eventually, least absolute shrinkage and selection operator (LASSO) logistic regression were applied to further dimensionality reduction and selection with log(λ) value of −3.022. 20 radiomics features were selected and divided into two groups: Group 1 (first order statistics features) and Group 2 (texture features). Details of selected features and coefficients are shown in Figure 4C.

### Model construction and validation

Based on the selected features, six machine learning models were established with six classifiers, k-NearestNeighbor (KNN), Support Vector Machine (SVM), eXtreme Gradient Boosting (XGB), Random Forest (RF), Logistic Regression (LR) and Decision tree (DT). After comparison with those models, XGBoost was selected as the most robust model. Considering its feature importance as radscore, the combined model was constructed by incorporating clinical risk factors with multivariate logistic regression. Beyond that, the radscore was chosen to construct a nomogram incorporating indwelling time. In order to improve those models, Hyper-parameter tuning was then performed using grid-search nested CV and selected based on its ROC-AUC. Parameter information was presented in Supplementary material S2.

The above models were conducted on the training cohort. Both were validated on the internal and external validation cohorts by importing relatively selected features in the R software package (version 4.3.1). Accuracy, sensitivity, specificity, area under the curve (AUC), decision curve analysis (DCA) and calibration curve were calculated in the software package R (version 4.3.1) and python (version 3.7) to evaluate the predictive performance of models. The procedures of model construction and validation are displayed in Figure 2.

## Results

### Baseline characteristics

The detailed characteristics of patients are collated in Table 1. The incidence rate of encrustation in SYSU5H and ZHP are 37.4% (101 of 270) and 38.1% (32 of 84), respectively, corresponding to Dyer’s viewpoint (2).

According to the grading system proposed by Javier, the distribution of encrustation locations are kidney (K: 4 of 101), ureter (U: 60 of 101), bladder (B: 24 of 101), kidney and ureter (KU: 0 of 101), kidney and bladder (KB: 7 of 101), ureter and bladder (UB: 5 of 101), kidney and ureter and bladder (KUB: 1 of 101) in SYSU5H and ureter (U: 18 of 32), bladder (B: 11 of 32), ureter and bladder (UB: 2 of 32), kidney and ureter and bladder (KUB: 1 of 32) in ZPH, respectively (23).

As we can see in Table 1, the indwelling time of ureteral stent placement in the encrustation cohort [median: 35 days; interquartile range (IQR): 28–55] is longer than in the non-encrustation cohort (median: 28 days; IQR: 27–32) in SYSU5H. A statistically significant difference was detected between the encrustation and non-encrustation cohorts regarding the indwelling time of ureteral stent placement and radiomics score (*p* < 0.01, *p* < 0.01, respectively). The average KUB scores are 2.65 ± 0.99 and 2.25 ± 1.41 in SYSU5H and ZPH, respectively. 88 and 85.1% of encrustations from SYSU5H were recognized as low or median degrees and divided into Grade 1 or 2 in the FECal grade and Grade A or B in the V-GUES classification, respectively. Similar outcomes were found in ZPH.

### Feature selection

Least absolute shrinkage and selection operator (LASSO) logistic regression was chosen to reduce dimension and select high-throughput features, characterized by variable selection and regularization while fitting the generalized linear model (24). The most beneficial 20 predictive features were selected from the overall 1,409 extracted features, and a histogram presented their coefficients in Figure 4C. In the study, the radiomics signature consists of 6 first-order features and 14 texture features. And textural features included five types: the gray level run-length matrix (GLRLM, *n* = 5), gray level size zone matrix (GLSZM, *n* = 2), the gray level co-occurrence matrix (GLCM, *n* = 5), gray level dependence matrix (GLDM, *n* = 1), neighboring gray tone difference matrix (NGTDM, *n* = 1). The details of features are illustrated in Supplementary material S3.

### Models construction and validation

**Figure 5:**
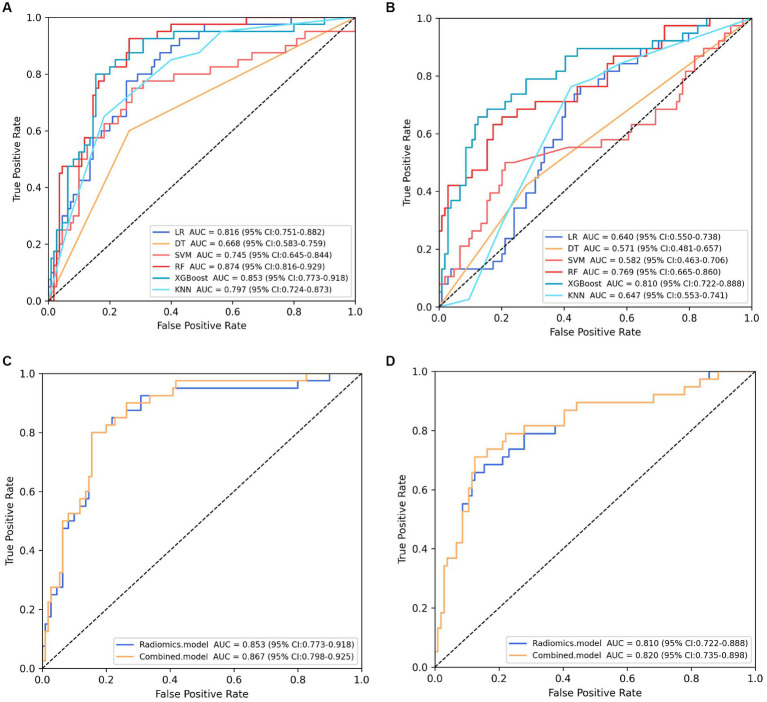
Performance comparison of models. **(A)** **(B)** showing the area under the curve (AUC) of six machine learning models in the internal and external validation cohort, respectively. Classifier RF and XGBoost constructed the most robust model in the internal and external validation cohort, respectively. **(C)** **(D)** presenting the performance of the selected radiomics model (XGBoost model) and combined model in the internal and external validation cohort, respectively.

The training cohort built six machine-learning models using different classifiers (KNN, SVM, XGB, RF, LR, DT) and its performance was summarized in Table 2. XGBoost model was the most robust radiomics model with an AUC of 0.810 (95%CI, 0.722–0.888) in the external validation cohort. Based on the feature importance of radiomics model, the combined model was established and presented a favorable identification ability of ureteral stent encrustation with an AUC of 0.999 (95%CI, 0.998–1.000). in the training cohort. For better application in clinical practice, a user-friendly nomogram was created (Figures 5 6).

**Table 2:** Performance of models.

	Model	AUC (95% CI)	Accuracy	Sensitivity	Specificity
Training cohort	LR	0.888 (0.854–0.920)	0.811	0.86	0.793
	DT	1.000 (1.000–1.000)	1	1	1
	SVM	1.000 (1.000–1.000)	1	1	1
	RF	0.994 (0.988–0.998)	0.96	0.968	0.957
	XGBoost	0.999 (0.998–1.000)	0.98	1	0.973
	KNN	0.962 (0.943–0.977)	0.897	0.849	0.914
	Combined	0.999 (0.998–1.000)	0.98	1	0.973
Internal validation cohort	LR	0.816 (0.751–0.882)	0.72	0.8	0.691
	DT	0.668 (0.583–0.759)	0.7	0.6	0.736
	SVM	0.745 (0.645–0.844)	0.733	0	1
	RF	0.874 (0.816–0.929)	0.8	0.825	0.791
	XGBoost	0.853 (0.773–0.918)	0.833	0.8	0.845
	KNN	0.797 (0.724–0.873)	0.773	0.65	0.818
	Combined	0.867 (0.798–0.925)	0.833	0.8	0.845
External validation cohort	LR	0.640 (0.550–0.738)	0.62	0.553	0.644
	DT	0.571 (0.481–0.657)	0.641	0.421	0.721
	SVM	0.582 (0.463–0.706)	0.732	0	1
	RF	0.769 (0.665–0.860)	0.768	0.632	0.817
	XGBoost	0.810 (0.722–0.888)	0.768	0.711	0.788
	KNN	0.647 (0.553–0.741)	0.662	0.289	0.798
	Combined	0.820 (0.735–0.898)	0.782	0.763	0.788

AUC, area under the curve; LR, Logistic Regression; SVM, Support Vector Machine; DT, Decision tree; KNN, k-NearestNeighbor; XGB, eXtreme Gradient Boosting; RF, Random Forest.

Validation was conducted in two independent validation cohorts to assess the models’ performance, and specific results were presented in Table 2. After comparison, the performance of the combined model was superior to others in internal validation and external validation, with ACU of 0.867 (95%CI, 0.798–0.925) and 0.820 (95%CI, 0.735–0.898), an overall accuracy of 78.2%, an sensitivity of 76.3%, an specificity of 78.8%, respectively. And calibration curve and decision curve analysis was presented in Figure 7, demonstrating good clinical utility.

**Figure 6:**
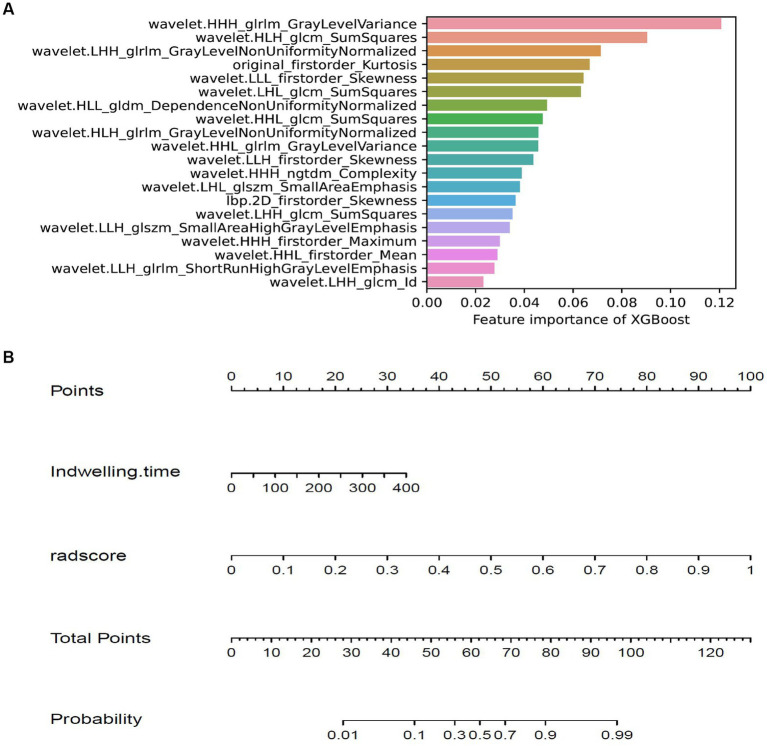
Feature importance of XGBoost model and the nomogram. **(A)** showing the importance of features in building XGBoost model. **(B)** The nomogram was developed based on the feature importance of XGBoost model (radsocre) and clinical risk factor (indwelling time).

## Discussion

In the present study, we developed seven radiomics models by analyzing non-contrast CT radiomics features extracted from an independent cohort of consecutive patients with ureteral stent placement. Furthermore, the validation of seven models performed in an internal and an independent external cohort presented a high accuracy rate in identifying ureteral stent encrustation. So far, no literature has been reported regarding identifying ureteral stent encrustation based on radiomics and machine learning methods, and our study is the first of its kind.

**Figure 7:**
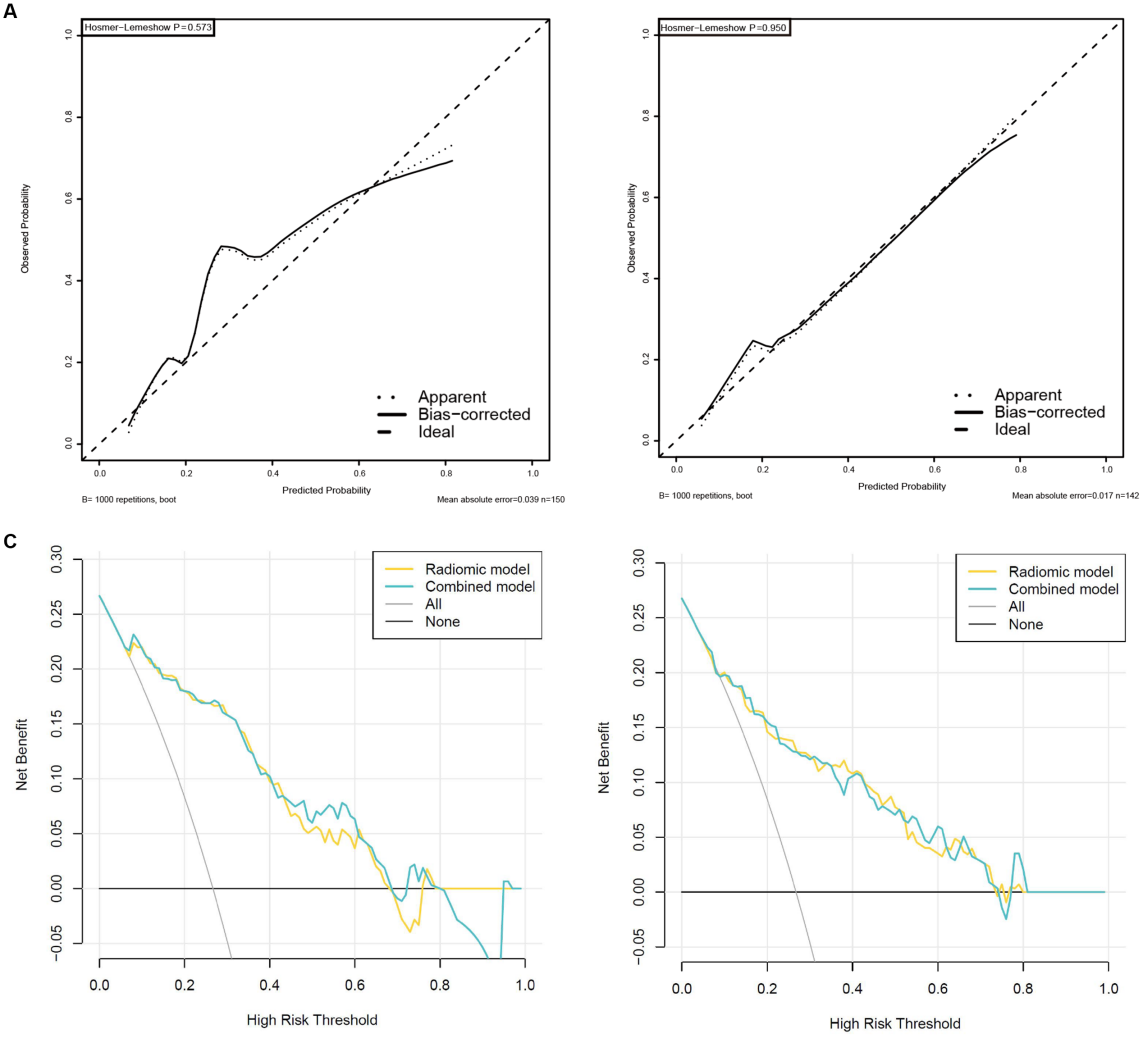
High-level calibration curves and decision curve analyses of the radiomics and combined model. **(A)** Internal validation cohort. **(B)** External validation cohort. **(C)** Internal validation cohort. **(D)** External validation cohort.

Ureteral stent encrustation gives rise to infection, obstruction, removal failure, etc. (2, 25). However, identifying ureteral stent encrustation on non-contrast CT is challenging, which troubles urologists and radiologists for a long time. Once the ureteral stent has encrusted, it could be challenging to remove it through only cystoscope with grasping forceps in the outpatient setting (26, 27). Because encrustations contribute to congestion in the urethral canal and scratch urethral mucosa, generating additional implications during the removal procedure. Thus, patients usually suffer physically and financially, including a second removal surgery in OR and extra medical expenditure.

Endourological management of ureteral stent encrustation remains technically and strategically challenging (28). Multimodal surgery is generally required. A systematic review on behalf of the EAU YAU Urolithiasis Group revealed that 27% of the encrusted stents require a combined surgery, followed by 24% of URS alone or 19% of SWL alone as a single surgery (29, 30). Because of the short indwelling time, the encrustations included in our study are principally low or median degrees by three evaluation tools, which are not easily identifiable. The combined model enables identification of suspicious encrustation with high accuracy, which assists urologists in taking a single timely surgery and prevents further aggravation (21, 31).

Urine leaves a layer of bacterial film on the surface of the ureteral stent, which provides the conditions to deposit mineral substances necessary for encrustation formation (32–34). Properties of materials vary between stents and encrustations, and the information of density or gray levels reflected on noncontract CT images can be absorbed precisely, but not by humans. Radiomics features extracted from the above two materials confirmed that point. That is part of why urologists and radiologists’ single visual identification of morphological differences often misses encrustations (35).

Regarding the encrusted ureteral stent we collected, 12 encrustations, 87 encrustations, and 51 encrustations occur at the stent’s renal, ureteral, and vesical portions, respectively. Interestingly, we found that severe encrustations tend to preferentially appear at the renal and vesical portions of the stent. A thin encrustation layer was usually attached to the circumference of the ureteral section, consistent with the deposition characteristics of encrustation reported in the literature (2, 10). For one more reason, we observed that encrustations at the ureteral portion tend to be scratched off.

Non-invasion and reproducibility are the prominent superiorities of radiomics models based on a mass of radiomics features extracted from non-contrast CT. We only need to segment VOIs and the machine-learning model enables us to tell if it is encrusted. However, not all radiomics models are characterized by excellent accuracy, specificity and sensitivity in actual medical practice due to CT data acquired from different CT scan machines and diverse settings (36, 37). For this reason, our radiomics model was constructed with relatively large positive data and verified with an independent group at an external medical institution, confirming that our machine learning models are robust and practical in clinical procedures.

20 radiomics features have been selected from 1,409 radiomics features extracted from 641 VOIs with Wilcoxon test, Spearman Correlation Analysis and LASSO. Interestingly, texture features, describing the spatial arrangement relationship between the gray levels of image voxels (38), comprised most of all features, reflecting the difference in physical properties between stent and encrustation. That is potentially part of why the radiomics model performs efficiently with an AUC of 0.810 (95%CI, 0.722–0.888). We tried to work with only one clinical factor, the indwelling time, and also established a clinical model in Supplementary materials S4 and 5. Unfortunately, it can hardly possess identification ability with poor outcome of ACU, DCA and calibration curve.

There are some limitations in the present study. First, it is retrospective research, and prospective research should be carried out to support the conclusion. Second, although we involved a few cases from another medical institution as an external set, multicenter cases are still needed for further validation.

In conclusion, we developed and validated a robust combined model and a nomogram fed by many radiomics features extracted from noncontract CT. These offer a high recognition of ureteral stent encrustation. An independent external cohort proved its robustness and excellent identification rate. In actual clinical procedures, it assists urologists and radiologists in diagnosing ureteral stent encrustation and reduces the needless loss of patients financially and physically. In the machine learning and artificial intelligence field, we explored uncharted territory outside of tumor and lithiasis (39). It is the first attempt to apply radiomics to identify ureteral stent encrustation, which bursts out much potentiality in urology.

## Data availability statement

The original contributions presented in the study are included in the article/Supplementary material, further inquiries can be directed to the corresponding authors.

## Ethics statement

The retrospective study was approved by the Ethics Review Committee of the Fifth Affiliated Hospital of Sun Yat-sen University, and the requirement for informed consent was waived concurrently.

## Author contributions

JQ: methodology, software, validation, formal analysis, data curation, and writing – original draft. MY: methodology, validation, writing – original draft, and visualization. HW: software, formal analysis, and visualization. ZL: resources, investigation, and data curation. GW: resources and data curation. XW, QG, and HH: data curation. JC: conceptualization, writing – review and editing, and supervision. YD: conceptualization, writing – review and editing, supervision, and project administration. All authors contributed to the article and approved the submitted version.

## Conflict of interest

The authors declare that the research was conducted in the absence of any commercial or financial relationships that could be construed as a potential conflict of interest.

## Publisher’s note

All claims expressed in this article are solely those of the authors and do not necessarily represent those of their affiliated organizations, or those of the publisher, the editors and the reviewers. Any product that may be evaluated in this article, or claim that may be made by its manufacturer, is not guaranteed or endorsed by the publisher.

## Footnotes

^1^ http://radcloud.cn/

## References

[1] Tomer N Garden E Small A Palese M. Ureteral stent encrustation: epidemiology, pathophysiology, management and current technology. J Urol. (2021) 205:68–77. doi: 10.1097/JU.0000000000001343, PMID: 32856981

[2] Dyer RB Chen MY Zagoria RJ Regan JD Hood CG Kavanagh PV. Complications of ureteral stent placement. Radiographics. (2002) 22:1005–22. doi: 10.1148/radiographics.22.5.g02se081005 12235330

[3] El-Faqih SR Shamsuddin AB Chakrabarti A Atassi R Kardar AH Osman MK. Polyurethane internal ureteral stents in treatment of stone patients: morbidity related to indwelling times. J Urol. (1991) 146:1487–91. doi: 10.1016/S0022-5347(17)38146-6 1942324

[4] Frant M Dayyoub E Bakowsky U Liefeith K. Evaluation of a ureteral catheter coating by means of a BioEncrustation in vitro model. Int J Pharm. (2018) 546:86–96. doi: 10.1016/j.ijpharm.2018.04.023, PMID: 29752980

[5] Wiseman O Ventimiglia E Doizi S Kleinclauss F Letendre J Cloutier J. Effects of silicone Hydrocoated double loop ureteral stent on symptoms and quality of life in patients undergoing flexible Ureteroscopy for kidney stone: a randomized multicenter clinical study. J Urol. (2020) 204:769–77. doi: 10.1097/JU.0000000000001098, PMID: 32364838

[6] Zhang Y He J Chen H Xiong C. A new hydrophilic biodegradable ureteral stent restrain encrustation both in vitro and in vivo. J Biomater Appl. (2021) 35:720–31. doi: 10.1177/0885328220949376, PMID: 32799701

[7] Mohammadi A Rakebi MM Gholamnezhad M Ahmadi Pishkuhi M Aghamir SMK. Does potassium citrate administration change the type and composition of encrusted material on double-J stent compared to primary stone? Int Urol Nephrol. (2021) 53:1797–803. doi: 10.1007/s11255-021-02891-x, PMID: 34050877 PMC8164059

[8] Torrecilla C Fernández-Concha J Cansino JR Mainez JA Amón JH Costas S. Reduction of ureteral stent encrustation by modulating the urine pH and inhibiting the crystal film with a new oral composition: a multicenter, placebo controlled, double blind, randomized clinical trial. BMC Urol. (2020) 20:65. doi: 10.1186/s12894-020-00633-2, PMID: 32503502 PMC7275439

[9] Bernasconi V Tozzi M Pietropaolo A De Coninck V Somani BK Tailly T. Comprehensive overview of ureteral stents based on clinical aspects, material and design. Cent European J Urol. (2023) 76:49–56. doi: 10.5173/ceju.2023.218, PMID: 37064263 PMC10091895

[10] Singh I Gupta NP Hemal AK Aron M Seth A Dogra PN. Severely encrusted polyurethane ureteral stents: management and analysis of potential risk factors. Urology. (2001) 58:526–31. doi: 10.1016/S0090-4295(01)01317-6, PMID: 11597531

[11] Duty B Okhunov Z Okeke Z Smith A. Medical malpractice in endourology: analysis of closed cases from the state of New York. J Urol. (2012) 187:528–32. doi: 10.1016/j.juro.2011.10.045 22177166

[12] Vanderbrink BA Rastinehad AR Ost MC Smith AD. Encrusted urinary stents: evaluation and endourologic management. J Endourol. (2008) 22:905–12. doi: 10.1089/end.2006.0382, PMID: 18643720

[13] Lambin P Leijenaar RTH Deist TM Peerlings J de Jong EEC van Timmeren J. Radiomics: the bridge between medical imaging and personalized medicine. Nat Rev Clin Oncol. (2017) 14:749–62. doi: 10.1038/nrclinonc.2017.141, PMID: 28975929

[14] Marcu LG Forster JC Bezak E. The potential role of Radiomics and Radiogenomics in patient stratification by tumor hypoxia status. J Am Coll Radiol. (2019) 16:1329–37. doi: 10.1016/j.jacr.2019.05.018, PMID: 31492411

[15] Hameed BMZ Dhavileswarapu S Raza SZ Karimi H Khanuja HS Shetty DK. Artificial intelligence and its impact on urological diseases and management: a comprehensive review of the literature. J Clin Med. (2021) 10:1864. doi: 10.3390/jcm10091864, PMID: 33925767 PMC8123407

[16] Zheng J Kong J Wu S Li Y Cai J Yu H. Development of a non-invasive tool to preoperatively evaluate the muscular invasiveness of bladder cancer using a radiomics approach. Cancer. (2019) 125:4388–98. doi: 10.1002/cncr.32490, PMID: 31469418

[17] De Perrot T Hofmeister J Burgermeister S Martin SP Feutry G Klein J. Differentiating kidney stones from phleboliths in unenhanced low-dose computed tomography using radiomics and machine learning. Eur Radiol. (2019) 29:4776–82. doi: 10.1007/s00330-019-6004-7 30747299

[18] Tang L Li W Zeng X Wang R Yang X Luo G. Value of artificial intelligence model based on unenhanced computed tomography of urinary tract for preoperative prediction of calcium oxalate monohydrate stones in vivo. Ann Transl Med. (2021) 9:1129. doi: 10.21037/atm-21-965, PMID: 34430570 PMC8350703

[19] Zheng J Yu H Batur J Shi Z Tuerxun A Abulajiang A. A multicenter study to develop a non-invasive radiomic model to identify urinary infection stone in vivo using machine-learning. Kidney Int. (2021) 100:870–80. doi: 10.1016/j.kint.2021.05.031, PMID: 34129883

[20] Cui X Che F Wang N Liu X Zhu Y Zhao Y. Preoperative prediction of infection stones using Radiomics features from computed tomography. IEEE Access. (2019) 7:122675–83. doi: 10.1109/ACCESS.2019.2937907

[21] Lombardo R Tubaro A De Nunzio C. Ureteral stent encrustation: epidemiology, pathophysiology, management and current technology. J Urol. (2022) 207:248–9. doi: 10.1097/JU.0000000000001678, PMID: 33591807

[22] Arenas JL Shen JK Keheila M Abourbih SR Lee A Stokes PK. Kidney, ureter, and bladder (KUB): a novel grading system for encrusted ureteral stents. Urology. (2016) 97:51–5. doi: 10.1016/j.urology.2016.06.050, PMID: 27421780

[23] Rahman SA Walker RC Lloyd MA Grace BL van Boxel GI Kingma BF. Machine learning to predict early recurrence after oesophageal cancer surgery. Br J Surg. (2020) 107:1042–52. doi: 10.1002/bjs.11461 31997313 PMC7299663

[24] Lin TF Lin WR Chen M Yang TY Hsu JM Chiu AW. The risk factors and complications of forgotten double-J stents: a single-center experience. J Chin Med Assoc. (2019) 82:767–71. doi: 10.1097/JCMA.0000000000000161, PMID: 31356568 PMC13048003

[25] Ulker V Celik O. Endoscopic, single-session Management of Encrusted, forgotten ureteral stents. Medicina (Kaunas). (2019) 55:58. doi: 10.3390/medicina55030058, PMID: 30813602 PMC6473799

[26] Juliebø-Jones P Pietropaolo A Haugland JN Mykoniatis I Somani BK. Current status of ureteric stents on extraction strings and other non-cystoscopic removal methods in the Paediatric setting: a systematic review on behalf of the European Association of Urology (EAU) young academic urology (YAU) urolithiasis group. Urology. (2022) 160:10–6. doi: 10.1016/j.urology.2021.11.022, PMID: 34910924

[27] Tsaturyan A Faria-Costa G Peteinaris A Lattarulo M Martinez BB Vrettos T. Endoscopic management of encrusted ureteral stents: outcomes and tips and tricks. World J Urol. (2023) 41:1415–21. doi: 10.1007/s00345-023-04361-8, PMID: 37024556

[28] Massella V Juliebø-Jones P Pietropaolo A Beisland C Somani BK. Outcomes associated with the Endourological Management of Stent Encrustation: findings from a literature review on behalf of the EAU YAU urolithiasis group. Curr Urol Rep. (2023) 24:187–99. doi: 10.1007/s11934-023-01144-x, PMID: 36705840

[29] Juliebø-Jones P Pietropaolo A Æsøy MS Ulvik Ø Beisland C Bres-Niewada E. Endourological management of encrusted ureteral stents: an up-to-date guide and treatment algorithm on behalf of the European Association of Urology young academic urology urolithiasis group. Cent European J Urol. (2021) 74:571–8. doi: 10.5173/ceju.2021.0264, PMID: 35083079 PMC8771125

[30] Geraghty RM Davis NF Tzelves L Lombardo R Yuan C Thomas K. Best practice in interventional Management of Urolithiasis: an update from the European Association of Urology guidelines panel for urolithiasis 2022. Eur Urol Focus. (2023) 9:199–208. doi: 10.1016/j.euf.2022.06.014, PMID: 35927160

[31] Al KF Denstedt JD Daisley BA Bjazevic J Welk BK Pautler SE. Ureteral stent microbiota is associated with patient comorbidities but not antibiotic exposure. Cell Rep Med. (2020) 1:100094. doi: 10.1016/j.xcrm.2020.100094, PMID: 33205072 PMC7659606

[32] Buhmann MT Abt D Nolte O Neu TR Strempel S Albrich WC. Encrustations on ureteral stents from patients without urinary tract infection reveal distinct urotypes and a low bacterial load. Microbiome. (2019) 7:60. doi: 10.1186/s40168-019-0674-x, PMID: 30981280 PMC6462311

[33] Tunney MM Keane PF Jones DS Gorman SP. Comparative assessment of ureteral stent biomaterial encrustation. Biomaterials. (1996) 17:1541–6. doi: 10.1016/0142-9612(96)89780-8, PMID: 8853126

[34] Yoshida T Takemoto K Sakata Y Matsuzaki T Koito Y Yamashita S. A randomized clinical trial evaluating the short-term results of ureteral stent encrustation in urolithiasis patients undergoing ureteroscopy: micro-computed tomography evaluation. Sci Rep. (2021) 11:10337. doi: 10.1038/s41598-021-89808-x, PMID: 33990648 PMC8121799

[35] Thawani R McLane M Beig N Ghose S Prasanna P Velcheti V. Radiomics and radiogenomics in lung cancer: a review for the clinician. Lung Cancer (Amsterdam, Netherlands). (2018) 115:34–41. doi: 10.1016/j.lungcan.2017.10.015, PMID: 29290259

[36] Zhao B Tan Y Tsai WY Qi J Xie C Lu L. Reproducibility of radiomics for deciphering tumor phenotype with imaging. Sci Rep. (2016) 6:23428. doi: 10.1038/srep23428, PMID: 27009765 PMC4806325

[37] Joensuu H. Risk stratification of patients diagnosed with gastrointestinal stromal tumor. Hum Pathol. (2008) 39:1411–9. doi: 10.1016/j.humpath.2008.06.025 18774375

[38] Incoronato M Aiello M Infante T Cavaliere C Grimaldi AM Mirabelli P. Radiogenomic analysis of oncological data: a technical survey. Int J Mol Sci. (2017) 18:805. doi: 10.3390/ijms18040805, PMID: 28417933 PMC5412389

[39] Cicione A Stira J Tema G Franco A Ghezzo N Gravina C. Ureteral stent encrustation: evaluation of available scores as predictors of a complex surgery. Minerva urology and nephrology. (2023) 75:359–65. doi: 10.23736/S2724-6051.22.04999-0, PMID: 36286398

