# Supplementary figures and images for: Identifying ureteral stent encrustation using machine learning based on CT radiomics features: a bicentric study
Source: Front Med (Lausanne). 2023 Aug 2;10:1202486. doi: 10.3389/fmed.2023.1202486 (PMC10433756; doi:10.3389/fmed.2023.1202486)

## After batch calibration

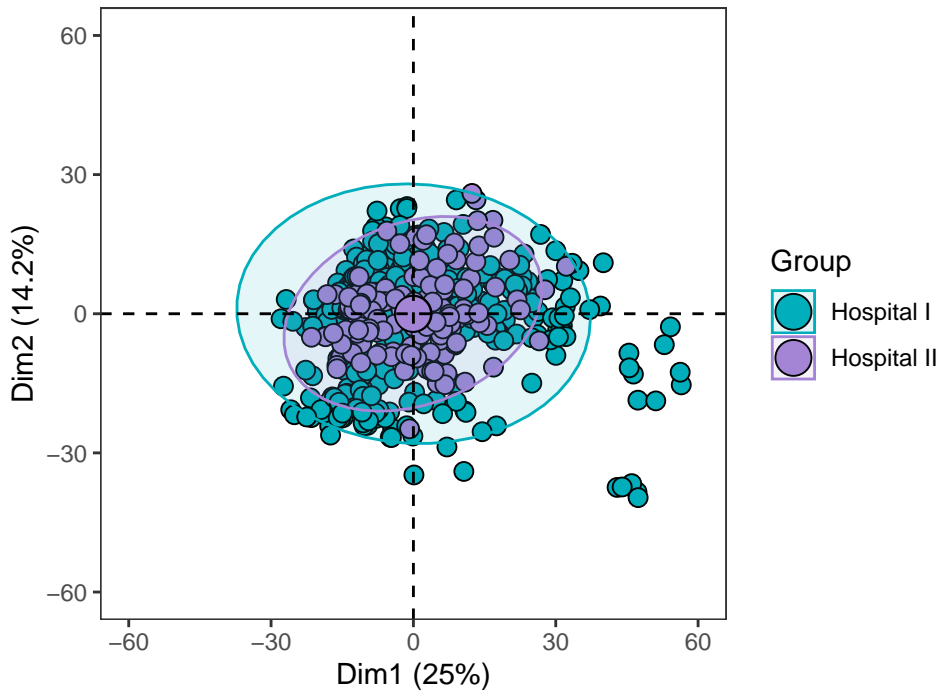

Supplement: Supplementary file 1 [file Data_Sheet_1.zip › Supplementary S1/After batch calibration.pdf]

Before batch calibration

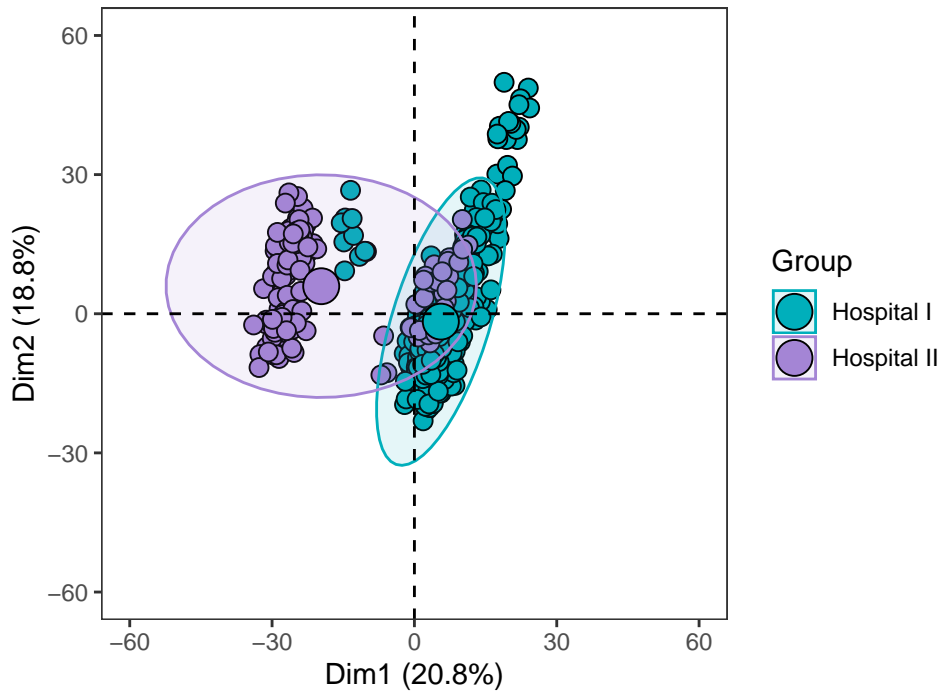

Supplement: Supplementary file 1 [file Data_Sheet_1.zip › Supplementary S1/Before batch calibration.pdf]
